# Psychosocial Working Conditions and Well-Being of Migrant Workers in Spain

**DOI:** 10.3390/ijerph17072547

**Published:** 2020-04-08

**Authors:** Francisco Díaz Bretones, Aditya Jain, Stavroula Leka, Pedro A. García-López

**Affiliations:** 1 School of Labour Relations and Human Resource, Universidad de Granada, 18071 Granada, Spain; pagarcia@ugr.es; 2 Nottingham University Business School, University of Nottingham, Nottingham NG8 1BB, UK; Aditya.Jain@nottingham.ac.uk; 3 Cork University Business School, University College Cork, T12 K8AF Cork, Ireland; stavroula.leka@ucc.ie; 4 Centre for Organizational Health and Development, School of Medicine, University of Nottingham, Nottingham NG8 1BB, UK

**Keywords:** migrant workers, psychosocial working conditions, well-being, Spain

## Abstract

This study examines the relationship beween employment and psychosocial working conditions and well-being of native and migrant workers in the working population of Spain. Data from the 7th Spanish Survey of Working Conditions was used to conduct a confirmatory factor analysis (n = 8508) to identify the main latent variables that influenced well-being. Using structural equation modeling and multivariate analysis, we found different patterns and perceptions of well-being and working conditions in these two groups. We discuss the reasons for these differences and suggest directions for further research in this area.

## 1. Introduction

Psychosocial hazards are discussed in guidance by key organizations (such as the International Labour Organization (ILO), World Health Organization (WHO), European Commission, etc.) as aspects of work organization, design and management that have the potential to cause harm on individual health and safety, as well as other adverse organizational outcomes such as sickness absence, reduced productivity or human error. They include several issues such as work demands, the availability of organizational support, rewards and interpersonal relationships. Psychosocial risk refers to the potential of psychosocial hazards to cause harm [1].

The available literature identifies at-risk groups in terms of employment conditions and personal characteristics, including gender, age and ethnicity, which highlight that migrant workers are more likely to be exposed to certain working and employment arrangements [2] that may place them at higher risk of future ill health. However, few studies have compared the relationship between employment and psychosocial working conditions and well-being in native versus migrant workers. The current study is innovative, as it aims to address this gap in the literature by comparing these groups in the working population of Spain. It does so using a large, representative sample of the Spanish population and employing robust statistical analysis to examine the relationship between employment and psychosocial working conditions and well-being in these groups of workers. The next sections provide an overview of the migrant workforce in the EU and Spain, review studies on the working conditions and well-being of migrant workers and present the conceptual framework adopted in this study.

### 1.1. The Migrant Workforce

According to ILO estimates, there are 150.3 million migrant workers in the world [3]. Historically, seeking employment has been a key factor in the flow of peacetime migration, with labor demands for expanding national economies, income inequalities among countries, and processes of economic integration all being contributory factors [4]. Spain received a large influx of migrants from various countries in the new millennium due to the country’s rapid economic growth and the demand for low-skilled labor. These were mainly migrants from Latin American countries and the Maghreb (North Africa) due to linguistic similarities and geographical proximity, respectively [5]. Their total number rose from 2% of the general population in 1998 to 12.2% in 2010 [6].

However, following the global 2008 economic crisis, the Spanish economy entered a period of recession, which produced a contraction in the labor market and a reduction in the number of migrant workers. Currently, 4,601,272 immigrants live in Spain (according to 2016 data from the Permanent Immigration Observatory) [7] out of a total population of 46,438,442 inhabitants, amounting to 9.91% of the total population. According to 2019 data from the Spanish National Statistics Institute, the 10 largest groups of migrants (foreign nationals) in Spain were from Morocco (682,022), Romania (673,592), UK (284,987), Italy (221,368), China (183,491), Colombia (159,563), Germany (138,777), Ecuador (139,441), Bulgaria (125,005) and Venezuela (91,131) [8]; in other words, countries from Europe, the Maghreb and Latin America.

### 1.2. Working Conditions and Well-Being of Migrant Workers

Migrant work has long been characterised as being low-paid and precarious, with workers reporting higher job insecurity and fewer entitlements than their native counterparts [9,10]. In addition, there is evidence that migrant workers in precarious work tend to be tasked with more difficult jobs and jobs that native workers do not want [4].

Research also clearly highlights that migrant workers face discrimination and bullying compared to local workers [4,11,12,13]. The implications of this include increased job dissatisfaction and frustration, making workers vulnerable to work-related stress [14,15]. According to Ronda-Pérez et al. [16], migrant workers are more likely to suffer from exposure to psychosocial risks at work due to a lack of social and family support in the country of relocation. Lack of support from co-workers and supervisors is also common, mainly because of cultural and language barriers [17,18]. There is also some evidence that suggests that migrant workers constitute one of the most vulnerable social groups exposed to poor employment and working conditions, especially during times of economic recession [19]. This is due to increased job insecurity, which has been found to be associated with poor employee well-being [20,21], including mainly mild-to-moderate depressive and anxiety disorders [22].

Tentative evidence on migrant worker well-being indicates that migrant workers have poorer health and well-being than native workers. Some studies link migration with a greater predisposition to depression (including major depression), depressive symptoms [23,24] and anxiety disorders [25]. Migrant workers whose social relationships with other co-workers are poor (i.e., when receiving low social support and/or being victims of social discrimination) have been found to have significantly higher levels of psychological distress and mental health problems. Moreover, those experiencing both high job demands and low decision latitude have been found to be under increased risk of long-term illness and mental health problems [18].

However, the mere fact of being a migrant does not presume a tendency to manifest depression, anxiety or somatic symptoms. Some studies have shown opposite findings. For example, Lindert et al. [26] found similar prevalence rates of depression and anxiety among labor migrants to the general US population. This has been attributed to the “healthy migrant hypothesis” [27], which postulates that migrant workers tend to be younger and healthier when migrating to a new country and that over time, with exposure to poor living and working conditions, these effects diminish. An alternate explanation for better migrant health could be due to underreporting, lack of awareness and help-seeking or the desire to appear healthy in order to remain economically valuable to an employer [28].

Nevertheless, it should be acknowledged that the main limitations of most studies on the working conditions of migrant workers are both lack of generalizability due to research conducted with non-representative samples [16] and a primary focus on ill health or access to healthcare, with limited considertation of working conditions [16,29].

### 1.3. Conceptual Framework

**Figure 1:**
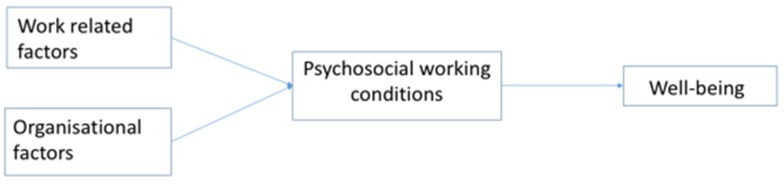
Study conceptual framework.

This paper aims to expand the existing literature by comparatively examining the relationship between employment (organizational and work-related) factors, psychosocial working conditions and well-being in migrant and native workers in Spain. The study uses the conceptual framework presented in Figure 1.

More specifically, in line with the existing literature, our study hypothesizes that specific organizational factors (such as occupational group or company size) and work-related factors (such as work contract or working hours) will be related to the psychosocial working conditions (e.g., job insecurity, workload, social support or autonomy) of both migrant and native workers in Spain, which will in turn be related to worker well-being. This framework is in line with both the European Framework for Psychosocial Risk Management (PRIMA-EF) [30] and the job demands-resources model [31], which theorize that characteristics of organizations and the way work is organized are related to job characteristics and can affect a range of outcomes, including employee well-being and organizational performance. These relationships are tested using the 7th Spanish Survey of Working Conditions that includes data collected between October 2011 and February 2012.

## 2. Materials and Methods

### 2.1. Data and Sample

The Spanish Survey of Working Conditions (Encuesta Nacional de Condiciones de Trabajo—ENCT) is a national cohort study designed and conducted by the Ministry of Employment (Spain) to analyze exposure to various occupational hazards and the factors in the work environment that influence the health of workers. The Spanish Survey of Working Conditions is included in the National Statistical Plan, in accordance with the Laws 4/1990 and 13/1996 of the Kingdom of Spain, ensuring ethical data collection, storage and handling of data. The data confidentiality is assured by the Law 12/89 of the Government Statistic Act that guarantees that the data provided are covered by statistical confidentiality, avoiding its misuse in all cases. In our study, we use data from the 7th Spanish Survey of Working Conditions (the most recent version available to date), which encompasses data of the fieldwork performed between October 2011 and February 2012. The geographical scope of the survey was the entire Spanish territory.

The ENCT is based on population register data as a directory to extract the sample of dwellings to be used for the interviews, combining probabilistic with other characteristics from quota sampling. The population scope is defined as the employed population aged 16 and over, from all economic activities, residing in family housing. The collection of data was carried out through face-to-face structured interviews in family residences. In total, 22,312 homes were visited with a decline rate of 60.15%. The absence of the residents at their homes (24.3%), the lack of workers in the household (17.7%), refusal to cooperate (4.9%) and empty dwellings (4.3%) were among the main reasons for an initial decline to participate. The obtained sample was made up of 8892 people (8070 Spanish vs. 814 from other nationalities), aged between 16 and 64, from all economic activities. After excluding 384 non-responses (4.32%), our final study sample comprised of 8508 respondents (90.87% native workers: n = 7731 and 9.13% migrant/foreign workers: n = 777). These data are close to the population distribution in 2011, when there were 5,751,487 foreign nationals registered in Spain (12.29% of the Spanish population) [6].

This study uses the term “migrant worker” to refer to people whose country of origin is different to the country they are residing and working in. We therefore dichotomized the question “nationality” (Q60) into two answers (Spanish/Other nationality). A “Spanish worker” is therefore synonymous with a “native worker”, while a “migrant worker” is synonymous with a “foreign worker” for the purposes of this study. A descriptive analysis of nationalities indicated that the majority of the foreign workers came from America (43.3%) and other European countries (37.6%), whereas only 16.1% and 2.9% came from Africa and Asia, respectively. By country, the largest groups were born in Romania (13%), Morocco (12.9%), Ecuador (10.5%), Colombia (6.3%), Argentina (4.7%) and Peru (4.4%), representing 51.8% of the subsample.

### 2.2. Measures

The 7th Spanish Survey of Working Conditions (ENCT) has 62 questions classified into 14 categories: (a) employment status (questions 1 to 7); (b) information about the workplace (questions 8 and 9); (c) type of job (questions 10 to 14); (d) physical agents (questions 15 to 18); (e) chemical and biological contaminants (questions 19 to 25); (f) safety conditions (questions 26 and 27); (g) workplace design, workload and psychosocial factors (questions 28 to 33); (h) prevention organization (question 34); (i) working hours (questions 35 to 40); (j) preventive activities (questions 41 to 47); (k) information/training (questions 48 and 49); (l) violent behaviors at the workplace (question 50); (m) health hazards (questions 51 to 55); and (n) sociodemographic data (questions 56 to 62).

For the purpose of this study, the following items were selected:

*Organizational factors*: “Hired by company or subcontractor?” (Q5), “Main activity of your company?” (Q6) and “How many people work in your company?”(Q7).

*Work-related factors*: “Type of employment contract?” (Q3), “Which type of job do you have?”(Q11), “In which conditions do you work (isolation/cooperation with other workers)?” (Q12), “How long have you been working in your workplace?” (Q13) and “On average, how many hours a week do you work (excluding lunch time)?” (Q35).

*Psychosocial working conditions*: “To what extent are you annoyed or worried about the risk of losing your current work?” (Q55_18) (job insecurity was conceptualized as a health hazard in line with Probst and Jiang [20]). Other items selected for inclusion were: “In your workplace, how often can you receive help from your colleagues if you ask for it?” (Q30_1), “…Can you receive help from your superiors/bosses if you ask for it working at very high speeds?” (Q30_2), “…Do you have the chance of doing what you do best?” (Q30_3), “…Can you put your own ideas into practice?” (Q30_4), “…Do you have the feeling of doing something useful?” (customers, passengers, students, patients, etc.) (Q30_5), “…Can you learn new things?” (Q30_6), “…Do you have much work and feel overwhelmed?” (Q30_7), “In your workplace, how often can you receive help from your colleagues if you ask for it?” (Q31_1), “… Can you receive help from your superiors/bosses if you ask for it?” (Q31_2), “…Do you have the chance of doing what you do best?”(Q31_3), “…Can you put your own ideas into practice?” (Q31_4), “In your workplace, how often can you choose or change the order of the tasks?” (Q32_1), “….The method of work?” (Q32_2), “…The rhythm of work?” (Q32_3) and “…The distribution and/or length of your breaks?” (Q32_4).

Responses for questions on job design and psychosocial factors Q30 (7 items), Q31 (4 items) and Q32 (4 items) were collected on a Likert-type scale, coded (from 1 to 5) for Q30, where 1 means “always” and 5 “never” (except Q31 and Q32, which were reverse-coded). For these questions (Q30, Q31 and Q32), a factor analysis was performed to estimate the latent factors by entering all scale items into a principal component analysis and examining the unrotated factor solution (Harman’s single-factor test) in order to identify if common method variance (CVM) is a problem within the data. This analysis did not produce a single assigned factor, since the main factor only explained 32.2% of the total variance [32]. The use of latent factors, instead of individual measures, is more parsimonious, as it allows a more accurate modeling of the measurement error and better explanation of the contribution of each measure [33]. Standardized factor loadings for each of these measures also ranged from 0.48 to 0.86, and all were statistically significant at the minimum probability level of 0.001 [34]. Table 1 shows the factor analysis and latent factors obtained.

*Well-being:* “Could you tell me if you have any of the following health conditions? Stress, anxiety or nervousness: Stress, anxiety or nervousness” (Q54_B13) and “…Tiredness or exhaustion?” (Q54_B15).

*Sociodemographic data*: Age (Q56), gender (Q58), educational level, “Which is the highest official education level you have?” (Q59) and nationality (Q60).

The results show three main psychosocial factors: factor 1, labeled as “autonomy”, factor 2, labeled as “workload” and factor 3, labeled as “social support”. Factor 4 was excluded from the analysis, as it comprised of only one item (Q30_1) and, therefore, was not reliable in large samples [35], while item Q30_5 was excluded because it had low loading scores in each factor [36].

### 2.3. Data Analysis

To analyze the relationships between these variables, the first step in the analysis was to describe the main sociodemographic characteristics, both in the general sample and in our two subsamples (native and migrant workers). Secondly, and in order to examine the differences between native and foreign workers, we carried out a chi-square analysis of all organizational and work-related factors described above using the Statistical Package for the Social Sciences (SPSS) version 23 (IBM, Armonk, NY, USA).

After selecting the organizational and work-related variables, and for the purpose of estimating the associations between these variables and the latent factors identified in our conceptual framework (see Figure 1), a linear regression within a structural equation modeling (SEM) framework was conducted with the “Lavaan R Package” [37]. Conventional levels of acceptable model fit (goodness-of-fit (GFI) and comparative fit index (CFI) values over 0.85; root mean square error approximation (RMSEA) values >0.05) and a statistically significant minimum probability level of 0.001 were taken into consideration [38,39].

## 3. Results

### 3.1. Descriptive Statistics 

The sociodemographic variables collected in the panel, as well as the descriptive analysis of the general population and both subsamples (migrant and native workers), are presented in Table 2. The findings show that while the profiles of both migrant vs. native workers are very similar, migrant workers are younger, with over 70% of the sample under 44 years of age. Additionally, migrant workers have higher levels of job insecurity, more fixed-term work contracts (55% more) and less seniority in their workplace as compared to native workers.

Chi-square analysis was used to evaluate the sampling distribution of the organizational and work-related variables described above in both subsamples through several contingency tables with the variable “nationality”. The results, presented in Table 3, indicate that there are significant differences between native and migrant workers across most variables.

The correlation matrix, means and standard deviations for these study variables are presented in Table 4.

### 3.2. Multivariate Models

This study sought to determine the relationship between employment and psychosocial working conditions on the well-being of migrant and native workers in Spain based on the 7th Spanish Survey of Working Conditions. To do so, we built and validated a multivariate model, with four factors as independent latent variables and well-being as the dependent variable.

**Figure 2:**
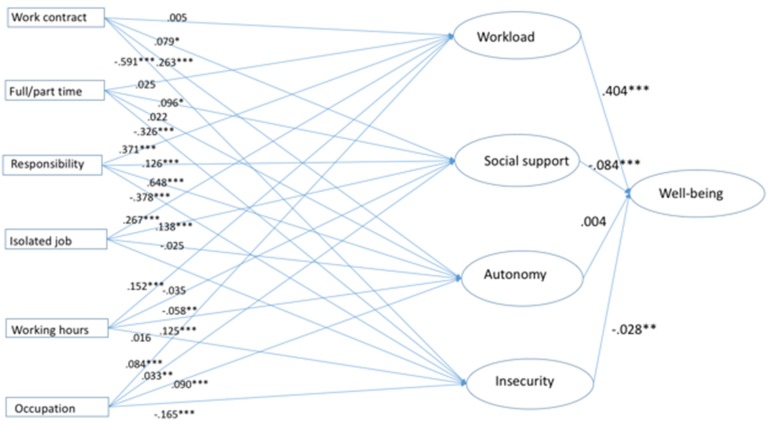
Structural equation model.

The fit of the structural equation model is determined by the fit indices that show the degree of concordance between the data predicted by the model and the observed data. The fit indices contribute to measure if the model fits well enough to provide a useful approximation to reality and a reasonable explanation of the data trends. According the criteria specified above, the model (see Figure 2) showed a very good fit based on the chi-square statistic: χ^2^(398) = 4062.75; *p* = 0.00. The fit statistical values for the model were acceptable: root mean square error approximation (RMSEA) = 0.052 (acceptable level < 0.08); *p* = 0.000 (acceptable level < 0.05), comparative fit index (CFI) = 0.913 (acceptable level > 0.85) and goodness-of-fit index (GFI) = 0.936 (acceptable level > 0.85).

The results highlight that the type of occupation, as well as responsibility over subordinates, were more strongly related with the latent variables, followed by working hours and type of contract. On the other hand, part-time work, as well as isolated work, showed the lowest contribution to the model. Regarding the four latent factors, there was a strong and statistically significant relationship in the case of “workload” and “social support” with well-being (with positive and negative association values, respectively). “Job insecurity” had a positive and significant association with well-being, whereas “autonomy” did not reach statistical significance.

Table 5 presents the results of the models for native and migrant workers. Model 1 examines the relationship between the study variables in migrant workers, whereas Model 2 does so in native workers. The evaluation of the two models in Table 5 suggests that, in the case of migrant workers, only workload was significantly associated with well-being, whereas social support and insecurity showed a nonsignificant association. A different pattern was observed in the case of Model 2 for native workers, where workload, social support and job insecurity were significantly associated with well-being. The latent factor “autonomy” was not associated with well-being in either of the two models.

In both models, associations were oberved between the latent variables and the different factors, with the exception of full/part-time work, which was not significantly related with any factor in Model 1 (migrant workers). Furthermore, the number of associations between the latent variables and their factors was much lower in Model 1 (migrant workers) than in Model 2 (native workers), which suggests that migrant workers report a weaker association between their well-being and organizational and work-related variables in comparison to native workers.

## 4. Discussion

This study aimed to expand the existing literature by comparatively examining the relationship between employment (organizational and work-related) factors and psychosocial working conditions and well-being in migrant and native workers in Spain. In line with the existing literature and our conceptual framework [30,31], we found that specific organizational factors (such as occupational group) and work-related factors (such as work contract, working hours and resposibiltiy to manage others) were related to psychosocial working conditions (job insecurity, workload, social support and autonomy) for both migrant and native workers in Spain, which, in turn, were found to have a strong association with worker well-being.

However, the results show different patterns and perceptions of well-being and working conditions in the two groups. In general, we found that migrant workers reported better well-being in comparison with the native population. While the literature suggests that migrant workers often report poorer health and well-being as compared to native workers (e.g., [21,22,23,24,40]), other studies highlight that this may not always be the case. For instance, in a follow-up survey conducted in 2008 and 2011 amongst migrant workers in Spain, Robert and collegues [41] found that there was an increase in poor mental health among immigrant workers who experienced deterioration in their employment conditions, probably influenced by the economic crisis. They, however, also found a decreased risk among those who attained their registration under the Spanish Social Security system. It is possible that a number of migrant workers in our study sample attained registration and, hence, reported better well-being outcomes. This is also likey since the 2008 financial crisis led to a reduction in the migrant workforce [7,8], and those workers choosing to remain in Spain may have remained due to better prospects as compared to those who may have returned to their home countries. A review of mental health in the immigrant population in Spain also noted that, while work and psychosocial factors are crucial to the mental health of immigrants, the main results of the studies conducted are inconsistent due to the early stage of research in this field in Spain [42].

The findings from this study support the general evidence that migrant workers have worse working conditions than native workers (e.g., [10,19]). We also observed that migrant workers had more unstable contracts and much longer or shorter working days than native workers. Despite this, only workload was significantly associated with well-being for migrant workers. While workload, social support and job insecurity were significantly associated with well-being for native workers in the multivariate models. Other studies on migrant workers have shown that social support and job insecurity have an impact on their well-being (e.g., [17,18,19,20,43]); however, we did not find the same relationships, which may be due to the effects of the healthy migrant worker hypothesis and the negative impact of the 2008 economic crisis on Spanish/native workers.

Some authors (e.g., [27]) argue that migrant workers are often younger (and probably healthier) than the native population. In this study, a comparison of the average age of the subsamples of migrant and native workers indicates that migrant workers were younger, and most were under 44 years of age. Furthermore, the healthy migrant worker hypothesis states that mainly physically healthy people will embark on the “migration adventure” towards another country [44], whereas unhealthier individuals return home (salmon bias hypothesis). The findings from this study provide some support to the healthy migrant worker hypothesis.

In addition to the healthy migrant worker hypothesis, while interpreting the findings of this study, we should also consider other factors that could explain the findings in relation to the well-being of migrant workers. Perceptions of health and well-being are subjective and include aspects of the cultural background, such as behaviors, norms, values and experiences which can help explain these divergences [45,46,47]. Thus, whereas workload was found to be related to well-being in both subsamples, social support and job insecurity were only found to have a relationship with well-being in the native, and not in the migrant, sample. These findings might suggest that migrant workers possibly develop better coping strategies to deal with adverse working conditions than native workers, and are more resilient or report less ill-health due to their wish to keep looking healthy and productive for the economic system [48,49,50,51]. In addition, as indicated earlier, an alternate explanation for better migrant health could be due to underreporting, lack of awareness and help-seeking or the desire to appear healthy in order to remain economically valuable to an employer [28]. Furthermore, it is possible that the use of a more objective measure of well-being, rather that a self-report measure, might have revealed more similar results in native and migrant workers.

Finally, the negative impact of the 2008 economic crisis on Spanish/native workers needs to be considered when interpreting the findings. The 2008 economic crisis in Spain has been associated with a significant increase in physical demands in Spanish workers, which until then was common among foreign national workers but, following the recession, was equally affecting Spaniards [19,52]. This might explain why we saw little difference in the well-being of native and migrant workers but more demands being reported in both groups. Other authors—for instance, Lindert et al. [27] in a meta-analytic review—found similar prevalence levels in anxiety and depression between migrant workers and the general population.

### Limitations

Since this is a cross-sectional study, we cannot infer causality. Since both independent and dependent variables have been based upon self-reports, there is risk of circularity in the cross-sectional analysis. However, our model is based on a robust representative sample, and we feel that the analysis techniques employed allowed for several insightful conclusions to be reached. By using structural equation models, we have managed to develop a cohesive and comprehensive model that adds confidence to the reliability of our results.

Another limitation is the fact that the analysis was based on secondary data, meaning that we did not have direct control over the design of the survey. As a result, the measures used are not standardized; however, many were adapted based on the literature, and other studies that aimed to examine effects in Spain also used some of these items [19]. Furthermore, some of our variables were assessed using a single-item. The rationale for using the specific items to measure our proposed variables was based on a comprehensive literature review, and several statistical checks were performed to examine appropriateness and the structure and reliability of the developed composite measures.

## 5. Conclusions

To sum up, this study has enabled us to establish that migrant workers are exposed to more adverse psychosocial risks at work as compared to native workers in Spain. However, the findings also highlight a more complex relationship between psychosocial working conditions and worker well-being. This should be considered further in future studies and policy development initiatives.

International migration processes are an unstoppable fact in all countries, and with the emergence of more ethnically and culturally diverse labor markets, organizations and society at large will have to consider their implications. Therefore, further reseach on the working conditions and well-being of migrant workers is needed, which aims to unravel their relationship within the constantly changing context of work.

## Figures and Tables

**Table 1:** Standardized factor loadings for each measure.

	Factor 1	Factor 2	Factor 3	Factor 4	Uniqueness
Q30_1	.	.	.	0.493	0.362
Q30_2	.	0.671	.	.	0.361
Q30_3	.	0.708	.	.	0.481
Q30_4	.	0.696	.	.	0.679
Q30_5	0.245	0.349	.	.	0.564
Q30_6	.	0.635	.	.	0.722
Q30_7	.	0.616	.	.	0.173
Q31_1	.	.	0.695	.	0.231
Q31_2	.	.	0.725	.	0.207
Q31_3	0.475	.	.	.	0.358
Q31_4	0.639	.	.	.	0.347
Q32_1	0.853	.	.	.	0.199
Q32_2	0.856	.	.	.	0.206
Q32_3	0.856	.	.	.	0.193
Q32_4	0.789	.	.	.	0.313

**Table 2:** Descriptive analysis of sociodemographic variables among migrant workers and native workers.

Variable	Total Sample (n = 8508)	Migrant Workers (n = 7731)	Native Workers (n = 777)
Age years: mean (SD)	42.51 (10.58)	38.48 (9.71)	42.92 (10.56)
16 to 24 (%)	289 (3.4%)	325 (4.2%)	26 (3.4%)
25 to 34 (%)	1838 (21.6%)	2683 (34.7%)	157 (20.2%)
35 to 44 (%)	2774 (32.6%)	2783 (36.0%)	251 (32.3%)
45 to 54 (%)	2382 (28.0%)	1438 (18.6%)	225 (29.0%)
55 and more (%)	1217 (14.3%)	487 (6.3%)	117 (15.1%)
No answer (%)	9 (0.1%)	15 (0.2%)	1 (0.1%)
Gender			
Male (%)	4518 (53.1%)	3989 (51.6%)	414 (53.3%)
Female (%)	3990 (46.9%)	3742 (48.4%)	363 (46.7%)
Educational level			
Less than secondary education (%)	3786 (44.5%)	3208 (41.5%)	348 (44.8%)
Secondary education (%)	2323 (27.3%)	2269 (34%)	207 (26.6%)
Higher education (%)	2348 (27.6%)	1794 (23.2%)	218 (28%)
Other (%)	51 (0.6%)	101 (1.3%)	5 (0.6%)
Seniority years: mean (SD)	8.95 (10.01)	3.84 (6.69)	9.48 (10.15)
Company size			
Less than 10 workers (%)	2204 (25.9%)	2273 (29.4%)	198 (25.5%)
Between 11 and 49 workers (%)	2467 (29.0%)	1786 (23.1%)	231 (29.7%)
Between 50 and 249 workers (%)	2127 (25.0%)	1971 (25.5%)	194 (25.0%)
More than 250 workers (%)	1710 (20.1%)	1701 (21.9%)	154 (19.8%)
Work contract			
Indefinite (%)	6789 (79.8%)	5102 (66.0%)	631 (81.2%)
Fixed term (%)	1719 (20.2%)	2629 (34.0%)	146 (18.8%)
Working hours week: mean (SD)	38.53 (10.38)	38.40 (12.32)	38.54 (10.17)
<35 h (%)	1455 (17.1%)	1716 (22.2%)	129 (16.6%)
35–40 h (%)	5130 (60.3%)	3835 (49.6%)	477 (61.4%)
>40 h (%)	1838 (21.6%)	2111 (27.3%)	163 (21.0%)
No answer (%)	85 (1%)	70 (0.9%)	8 (1%)
Insecurity: mean (SD)		3.47 (1.55)	3.18 (1.58)
Workload: mean (SD)		2.84 (0.91)	2.98 (0.88)
Social support: mean (SD)		4.42 (1.50)	4.58 (1.32)
Autonomy: mean (SD)		3.11 (1.19)	3.45 (1.11)

**Table 3:** Chi-square analysis of organizational variables and latent factors between migrant and native workers.

Variable	*x* ^2^	*df*	*p*	Associated Level for Inmigrants
Full/part time job **	3.830	1	0.002	Part-time
Hired company/subcontractor	0.929	1	0.335	
Work contract **	82.628	1	0.000	Temporary/none
Responsibility **	59.184	1	0.000	No
Isolated or cooperative job **	10.259	1	0.001	Isolated
Working hours per week **	57.408	5	0.000	<20 h and >40 h
Sector (occupation) **	40.184	3	0.000	Services
Insecurity **	31.875	4	0.000	
Workload **	43.913	20	0.002	
Social support **	63.732	11	0.000	
Autonomy **	101.915	24	0.000	

Note: **p < 0.01.

**Table 4:** Means, standard deviation and intercorrelations of the variables used in this study (n = 8508).

*Variable*	M	SD	1	2	3	4	5	6	7	8	9	10	11	12	13	14	15
1. Well-being	1.84	0.33	1														
2. Workload	2.97	0.88	0.24 **	1													
3. Social support	4.56	1.34	−0.10 **	−0.10 **	1												
4. Autonomy	3.41	1.21	−0.08 *	−0.08 **	0.35 **	1											
5. Insecurity	3.19	1.58	0.04	0.07 **	−0.11 **	−0.16 **	1										
6. Work contract	1.80	0.40	0.04	0.01	0.03 *	0.14 **	−0.18 **	1									
7. Full/part-time	1.87	0.34	0.08 *	0.06 **	0.02	0.02	−0.05 **	0.14 **	1								
8. Responsiblity	1.29	0.45	0.05	0.03 **	0.31 **	0.40 **	−0.16 **	0.14 **	0.10 **	1							
9. Isolated job	1.33	0.47	0.06	0.13 **	−0.05 **	−0.06 **	0.02 *	−0.03 *	0.05 **	−0.01	1						
10. Working hours	38.57	10.31	0.15 **	0.11 **	0.07 **	0.06 **	−0.01	0.10 **	0.61 **	0.21 **	0.03 **	1					
11. Activity sector	3.45	0.94	0.05	0.03 **	0.02 *	0.06 **	−0.08 **	0.09 **	−0.11 **	−0.03 **	−0.05 **	−0.12 **	1				
12. Nationality	1.09	0.29	0.00	−0.05 **	−0.03 **	−0.09 **	0.06 **	−0.11 **	−0.03 *	−0.08 **	−0.03 **	−0.01	0.01	1			
13. Gender	1.46	0.50	−0.03	−0.02 *	−0.02	−0.05 **	−0.01	−0.02 *	−0.25 **	−0.16 **	−0.01	−0.26 **	0.23 **	0.01	1		
14. Educational level	5.06	2.02	0.11 **	0.14 **	0.05 **	0.14 **	−0.15 **	0.09 **	0.04 **	0.10 **	0.09 **	−0.05 **	0.25 **	0.01	0.10 **	1	
15. Age (years)	42.47	10.35	−0.06	−0.10 **	0.06 **	0.12 **	−0.14 **	0.22 **	0.09 **	0.16 **	−0.04 **	0.06 **	−0.01	−0.13 **	−0.02 *	−0.07 **	1

Note: **p < 0.01 and *p < 0.05.

**Table 5:** Model coefficients and the standard error for linear regressions in migrant workers (Model 1) and native workers (Model 2).

	Model 1		Model 2	
Variable	Coefficient	(SE)	Coefficient	(SE)
***Workload***				
Well-being	0.400 **	(0.072)	0.402 **	(0.020)
Work contract	−0.092	(0.099)	0.015	(0.038)
Full/part-time	−0.225	(0.160)	0.050	(0.054)
Responsibility	0.524 *	(0.207)	0.361 **	(0.039)
Isolated job	0.311 **	(0.102)	0.259 **	(0.030)
Working hours	0.152	(0.062)	0.153 **	(0.022)
Occupation	−0.024	(0.050)	0.095 **	(0.016)
***Social support***				
Well-being	−0.025	(0.064)	−0.092 **	(0.018)
Work contract	0.150	(0.095)	0.043	(0.035)
Full/part-time	0.098	(0.152)	0.083	(0.050)
Responsibility	0.108	(0.195)	0.114 **	(0.036)
Isolated job	0.161	(0.097)	0.133 **	(0.028)
Working hours	0.012	(0.059)	−0.032	(0.021)
Occupation	0.090	(0.048)	0.027	(0.015)
***Autonomy***				
Well-being	0.093	(0.060)	−0.005	(0.017)
Work contract	0.185 *	(0.091)	0.255 **	(0.035)
Full/part-time	−0.200	(0.146)	0.036	(0.050)
Responsibility	0.933	(0.191)	0.622 **	(0.037)
Isolated job	−0.134	(0.093)	−0.018	(0.028)
Working hours	0.008	(0.057)	−0.059 **	(0.021)
Occupation	0.124 **		0.085 **	(0.015)
***Insecurity***				
Well-being	0.047	(0.040)	0.029 **	(0.010)
Work contract	−0.413 **	(0.127)	−0.603 **	(0.051)
Full/part-time	−0.020	(0.204)	−0.351 **	(0.071)
Responsibility	−0.389	(0.263)	−0.364 **	(0.052)
Isolated job	0.136	(0.129)	0.011	(0.040)
Working hours	−0.060	(0.079)	0.144 **	(0.030)
Occupation	−0.087	(0.064)	−0.173 **	(0.021)

Note: **p < 0.01 and *p < 0.05
